# Chronic Inflammation Mediates the Association between Cortisol and Hyperglycemia: Findings from the Cross-Sectional Population-Based KORA Age Study

**DOI:** 10.3390/jcm10132751

**Published:** 2021-06-22

**Authors:** Hamimatunnisa Johar, Derek Spieler, Martin Bidlingmaier, Christian Herder, Wolfgang Rathmann, Wolfgang Koenig, Annette Peters, Johannes Kruse, Karl-Heinz Ladwig

**Affiliations:** 1 Department of Psychosomatic Medicine and Psychotherapy, University of Gießen and Marburg, 35392 Gießen, Germany; hamimatunnisa.johar@helmholtz-muenchen.de (H.J.); johannes.kruse@psycho.med.uni-giessen.de (J.K.); 2 German Center for Diabetes Research (DZD), München, 85764 Neuherberg, Germany; christian.herder@ddz.de (C.H.); peters@helmholtz-muenchen.de (A.P.); 3 Institute of Epidemiology, Helmholtz Zentrum München, 85764 Neuherberg, Germany; derek.spieler@uniklinik-freiburg.de; 4 Department of Psychosomatic Medicine and Psychotherapy, Universitätsklinikum Freiburg, Albert-Ludwigs Universität Freiburg, 79085 Freiburg, Germany; 5 Medizinische Klinik und Poliklinik IV, Klinikum der Ludwig-Maximilians-Universität München, 80336 Munich, Germany; martin.bidlingmaier@med.uni-muenchen.de; 6 German Diabetes Center, Institute for Clinical Diabetology, Leibniz Center for Diabetes Research at Heinrich Heine University Düsseldorf, 40225 Düsseldorf, Germany; 7 Division of Endocrinology and Diabetology, Medical Faculty and University Hospital Düsseldorf, Heinrich-Heine-University Düsseldorf, 40225 Düsseldorf, Germany; wolfgang.rathmann@ddz.de; 8 German Diabetes Center, Institute for Biometrics and Epidemiology, Leibniz Center for Diabetes Research at Heinrich Heine University Düsseldorf, 40225 Düsseldorf, Germany; 9 Deutsches Herzzentrum München DZHK (German Centre for Cardiovascular Research), Partner Site Munich Heart Alliance, 80636 Munich, Germany; wolfgang.koenig@uniklinik-ulm.de; 10 Institute of Epidemiology, Medical Biometry University of Ulm, 89081 Ulm, Germany; 11 Department of Psychosomatic Medicine and Psychotherapy, Klinikum Rechts der Isar, Technische Universität München, 81675 Munich, Germany

**Keywords:** cortisol, interleukin-6, HbA1c, psychological stress, mediation analysis

## Abstract

(1) Background: The study aimed to investigate the role of subclinical inflammation on the association between diurnal cortisol patterns and glycaemia in an aged population. (2) Methods: Salivary cortisol, interleukin-6 (IL-6) and glycated haemoglobin (HbA1c) were analysed in a sample of 394 men and 364 women (mean age = 5 ± 6.3, 65–90 years). The ratio of morning after awakening and late-night cortisol was calculated as an indication of diurnal cortisol slope (DCS). Multivariable regression models were run to examine whether IL-6 mediates the relationship between the DCS and glycaemia. The Sobel test and bootstrapping methods were used to quantify the mediation analyses. (3) Results: In comparison to normoglycaemic counterparts (*n* = 676, 89.2%), an increase in IL-6 concentrations, in individuals with hyperglycaemia (HbA1c ≥ 6.5%) (*n* = 82, 10.8%) (*p* = 0.04), was significantly associated with a flatter DCS. The link between flatter DCS and elevated HbA1c level was significant mediated by a heightened IL-6 level. Our results do not suggest reverse-directionality, whereby cortisol did not mediate the association of IL-6 with HbA1c. (4) Conclusions: In our sample, the relation between flatter DCS and hyperglycaemia was partly explained by IL-6 levels. The paradigm of subclinical inflammation-mediated cortisol response on glucose metabolism could have widespread implications for improving our understanding of the pathophysiology of type 2 diabetes mellitus.

## 1. Introduction

The role of psychological stress factors in the pathogenesis of type 2 diabetes is increasingly acknowledged [1,2], although the exact mechanisms through which psychosocial stress act on hyperglycaemia are not well understood. There is little doubt that impairments of the hypothalamic-pituitary-adrenal (HPA) axis plays a major role in the crosstalk between psychosocial stress and the metabolic disruption. Here, cortisol, a glucocorticoid (GC) hormone and key component of the HPA axis, exerts counterregulating effects on insulin via induction of hepatic gluconeogenesis and inhibition of the peripheral uptake of glucose [3].

Stress-induced disruption of the HPA axis can result either in glucocorticoid excess, presumably based on altered feedback regulation of cortisol, or, conversely, in a blunted glucocorticoid secretion, most likely due to an impaired glucocorticoid receptor sensitivity or decreased responsiveness to glucocorticoids, a phenomenon known as glucocorticoid resistance [4]. A mismatch between the level of the cortisol awakening reaction (CAR) in the morning and the nadir in the evening, leading to a flatter diurnal cortisol slope (DCS), is a valid indicator of impaired HPA axis functioning [5]. A meta-analysis of 80 studies recently evidenced that a flatter DCS was associated with poorer general health outcomes [6].

Besides its critical role in energy balance, GC contributes to immune regulation by shutting down inflammatory processes to prevent host destruction due to excessive immune activity under acute “healthy” stress conditions [7]. However, sustained psychosocial stress conditions may promote proinflammatory effects caused by impaired GC receptor sensitivity [8,9]. Furthermore, the coexistence of dysregulated HPA-axis and increased inflammation has been increasingly acknowledged in the pathogenesis of type 2 diabetes [10], although conclusive evidence is lacking.

However, the pathway that mediates the link between cortisol and impaired glucose metabolism is still unexplored. To advance our current understanding of the crosstalk between impaired HPA axis functioning and heightened inflammation on metabolic dysregulation, we applied the glycated haemoglobin (HbA1c) measurement as an indicator of hyperglycaemia in the present study. Therefore, the percentage of HbA1c in blood samples indicates how well blood glucose has been controlled over the preceding months, reflecting the cumulative glycaemic history. HbA1c is superior to diabetes diagnosis alone as it provides a reliable measure of glucose regulation as patients who received antidiabetic treatments may have normalized blood glucose levels. It is well-established that the dysregulated cortisol secretion pattern is associated with type 2 diabetes or increased in glycosylated haemoglobin (HbA1c) levels, as previously shown [11,12]. Meta-analytic results indicate that IL-6 is the most sensitive inflammatory marker that predicts subsequent diabetes in initially healthy samples [10], and concentrations of IL-6 are elevated in patients with type 2 diabetes [13]. We hypothesized that flattened DCS is associated with elevated HbA1c levels mediated by greater subclinical inflammation as measured by IL-6. Therefore, in this cross-sectional population-based study, we aimed to elucidate whether subclinical inflammation mediates the association between diurnal cortisol secretion patterns and increased HbA1c levels in a representative community-dwelling older men and women.

## 2. Materials and Methods

### 2.1. Study Setting and Population

The KORA (Cooperative Health Research in the Region of Augsburg)-Age study is a follow up examination of the participants (*n* = 4127, age ≥ 64 years) of the previous four MONICA/KORA Surveys in the Augsburg region, Southern Germany, which was conducted between November 2008 and November 2009 [14] with participation rates between 67% and 79%. From these, a randomly drawn sample of 1079 participants participated in a standardised telephone interview and extensive physical examinations at the study centre, including the collection of blood samples, anthropometric examination, and personal interview.

Salivary samples were available from 772 subjects (saliva sampling rate of 72%) [11]. After exclusion of participants with missing data on type 2 diabetes, cortisol, and IL-6, the final data set for the present analysis consisted of 758 participants (394 males and 364 females) aged 65–90 years (mean = 75 ± 6.3). A drop-out analysis of the excluded participants revealed no significant age and sex differences.

The study was approved by the Ethics Committee of the Bavarian Medical Association, and all participants provided written informed consent.

### 2.2. Biomarker Measurements

For salivary cortisol sampling, participants were individually instructed about the procedure with detailed written information (Salivette^®^ test kit, Sarstedt, Nümbrecht, Germany). At home, participants collected 3 saliva samples: in the morning after awakening (M1) while sitting in an upright position, 30 min after awakening (M2), and in the late night before bedtime, on a day that should not involve any special occasions, such as family celebration, travel, or a doctor’s visit. Exact times of responses were recorded by the participants. We did not enforce a specific day for sampling but instructed the study participants to provide the samples all on the same day to reflect normal daily settings. Otherwise, the saliva collection should be postponed to other days. Participants were instructed not to eat, drink, or brush their teeth 15 min before the sampling. Analysis of self-documented collection times revealed that 95% of the subjects had collected the M2 sample with less than 5 min deviation from the expected timeframe. Cortisol levels (ng/mL or nmol/L) were determined in duplicate using a luminescence immunoassay (IBL, Hamburg, Germany). The lower detection limit of this assay is 0.1 ng/mL (0.276 nmol/L), intra- and inter-assay coefficients of variation (CV) are below 6% and 9% at concentrations of 0.4 ng/mL (1.1 nmol/L) and 5.0 ng/mL (1.38 nmol/L), respectively. All of the available salivary cortisol samples were within the lowest detection limit of the assay. Some individuals (late-night salivary cortisol, LNSC: *n* = 16; morning after awakening, *n* = 21) were excluded due to insufficient saliva sample volume for analysis. We calculated the area under the curve (AUC), based on the trapezoid rule, using all available data from the three time points (*n* = 734). The mean of total hours awake was 15.5 (±1.5) h.

The diurnal cortisol slope (DCS) represents the degree of change in cortisol from morning to late-night over the waking day [5]. Therefore, the ratio of M1 and LNSC was calculated as an indication of DCS. While lower DCS values indicate flatter DCS patterns, high DCS values correspond to steeper DCS or healthy diurnal patterns. The distributions of DCS were split by the tertiles, and subjects were stratified into those with flatter, medium, or steeper DCS.

Serum cortisol, IL-6 and HbA1c measurements were obtained from a blood sample during the physical examination at the study centre in the morning. Serum cortisol (µg/dL) was measured using the LIAISON chemiluminescence immunoassay (DiaSorin, Dietzenbach) according to the manufacturer’s instructions with intra- and inter-assay CV below 12.4% and 4.4%, respectively. IL-6 levels (pg/mL or IU/mL) were assayed using the Quantikine HS ELISA, SS600B (R & D Systems, Abingdon, UK) (inter-assay CV = 7.4%, intra-assay CV = 6.8%). HbA1c (mmol/mol and percentages (%)) was quantified with a reverse-phase cation-exchange HPLC method using a Menarini–Arkray Analyzer HA-8160 (Menarini Diagnostics, Florence, Italy). Results of the Diabetes Control and Complications Trial which shown that tight blood glucose control was associated with a reduced risk of diabetic complications and, therefore, the American Diabetes Association recommends an HbA1c of <6.5% for people with diabetes [15]. Therefore, individuals with hyperglycaemia are identified as having HbA1c levels of ≥6.5% (48 mmol/mol).

### 2.3. Covariate Measurements

Information on covariates was obtained in standardized personal interviews, conducted by trained medical staff, and a self-administered questionnaire as described elsewhere in detail [16]. Low education was defined as <12 years of schooling. Someone who smoked cigarettes regularly or occasionally was considered as a current smoker. Alcohol consumption was rated as “daily”, “once or several times a week”, and “no”. To assess physical activity, participants were classified as ‘active’ during leisure time if they regularly participated in sports for at least 1 h per week; otherwise, they were considered ‘inactive’. Type 2 diabetes was self-reported by the participants in the self-administered questionnaire and verified from physicians and the use of antidiabetic medications. Multimorbidity was defined as the co-occurrence of more than two disease conditions based on the Charlson Comorbidity Index [14]. Body mass index (BMI) was calculated as weight (kg)/height^2^ (m²), which was assessed in a medical examination. Hypertension was defined as blood pressure ≥ 140/90 mmHg and/or current use of hypertensive medication. Total cholesterol (TC) and high-density lipoprotein cholesterol (HDL-C) in mmol/L were measured by enzymatic methods (CHOD-PAP, Boehringer Mannheim, Germany). Psychological variables included depressive symptoms (cut-off point >5 for mild or moderate depression using the 15-item German version of the Geriatric Depression Scale), anxiety (cut-off point ≥10 for high anxiety using the Generalized Anxiety Disorder-7), sleeping problems (based on interview questions concerning the difficulty initiating and maintaining sleep), and perceived stress from a stressful life event (assessed by a two-item instrument based on the Psychosocial Stress Questionnaire from the Interheart Study) as previously described [11].

### 2.4. Statistical Analysis

Participants were grouped based on hyperglycaemic status (HbA1c ≥ 6.5% (48 mmol/mol)) and DCS tertile distribution for descriptive data analysis. Therefore, sociodemographic, lifestyle, clinical, and psychosomatic characteristics were stratified by hyperglycaemic status in Table 1 and by DCS tertiles in Table 2. Bivariate associations of biomarker groups and continuous variables were tested using the Kruskal-Wallis test and χ2 test was used for categorical variables. In case of non-normality, tests were performed on log-transformed biomarker measurements. LS (least squared) means and 95% confidence interval (CI) of biomarker measurements were calculated. Differences between groups (in age and sex adjusted models) and differences between men and women (adjusted for age) were tested with generalized linear model (GLM) procedures. Geometric means (95% confidence intervals) of IL-6 levels were calculated based on the antilog of standard deviations of log means. Associations between cortisol and glycaemic groups were tested with age and sex-adjusted generalized linear model (GLM) procedures.

We conducted statistical analyses using the biomarkers’ continuous value, modelled as an increment of 1 standard deviation (SD) of the log-transformed values. We first examined the interaction between DCS and IL-6 (product term of DCS and IL-6) in linear regression models with HbA1c levels as the dependent variable. Model 1 was adjusted only for age and sex. Model 2 was further adjusted for awakening time, education level, physical activity, current smoker, alcohol consumption, and depressive symptoms, while Model 3 was additionally adjusted for BMI.

We evaluated the accuracy of the mediation effect using the traditional hypothesized method using linear regression analyses (Baron & Kenny, 1986). We started by defining the mediation model within a four-step framework described by Baron and Kenny [17]. Step 1 examined the relationship between diurnal cortisol slope (DCS) and IL-6 level (path b). Step 2 examined the relationship between IL-6 and HbA1c while controlling for DCS (path c). Step 3 examined the relationship between DCS and HbA1c (path a). Step 4 examined the relationship between DCS and HbA1c while controlling for the IL-6 levels (path a’). Path a is the total effect, path a’ is the direct effect, and path b x c is the indirect effect (i.e., flatter DCS is associated with IL-6 levels, leading to heightened HbA1c levels). Furthermore, the Sobel test was used to test the significance of a mediation effect. It provides means to determine whether the reduction in the effect of the independent variable, after including the mediator, is a significant reduction and therefore, whether the mediation effect is statistically significant [18]. We then repeated the mediation analyses with nonparametric bootstrap, with 1000 resamples, to obtain the proportion mediated, the magnitude of the average total effect, and the significance of the indirect effects [19]. As a validation to the salivary samples, we repeated the whole analyses using serum cortisol samples.

Descriptive and regression analyses were run in SAS version 9.4 (SAS Institute Inc., Cary, NC, USA). Mediation analyses were performed by using ‘Mediation’ package in R. The significance level was set at 0.05. The study description followed the STROBE (STrengthening the Reporting of OBservational studies in Epidemiology) guidelines for observational studies.

## 3. Results

### 3.1. Description of the Study Population

The present investigation includes 758 participants (52% males and 48% females) with an overall mean of HbA1c of 5.7%, SD ± 0.6 (39.1 mmol/mol, SD ± 6.2), among whom 11% (*n* = 82) were identified being in a hyperglycaemic state (HbA1c ≥ 6.5% (48 mmol/mol)). As shown in Table 1, individuals with hyperglycaemia were more likely to have higher HbA1c levels, have a flatter DCS (lower M1 to LNSC ratio), have higher IL-6 levels, be hypertensive, have higher BMI, consume less alcohol, have higher total/HDL cholesterol levels, and be affected by multimorbidity in comparison to the normoglycemia group. There were no significant differences between glycaemic groups in sociodemographic and psychological factors, overall cortisol output (AUC), serum cortisol levels, smoking status, and physical activity. In sex-stratified analyses, men with hyperglycaemia had higher BMI and higher total/HDL cholesterol levels while women with hyperglycaemia had higher IL-6 levels, consumed more alcohol, and suffered more frequently from hypertension.

Table 2 presents the study characteristics according to DCS groups. Individuals with a flatter DCS were more likely to be older, have lower overall cortisol output (AUC), higher HbA1c, and higher IL-6 levels compared to those with healthier (steeper) DCS profiles. No sex-specific associations between DCS and baseline risk factors were observed except that men with flatter DCS were more likely to suffer from comorbidities while women with a flatter DCS tended to be less physically active.

### 3.2. Association of Cortisol and IL-6 in Individuals with Normoglycemia and Hyperglycemia

**Figure 1:**
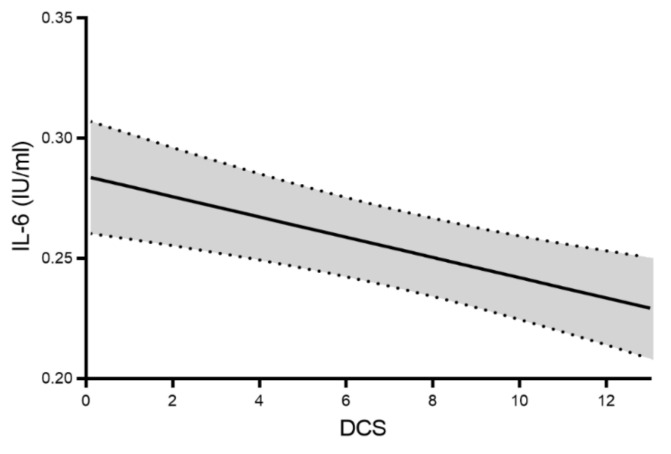
Linear regression analysis of the relationship between diurnal cortisol slope (DCS) and interluekin-6 (IL-6) levels (pg/mL). Higher DCS values indicate a steeper DCS. Abbreviations: Diurnal Cortisol Slope (DCS); Interleukin-6 (IL-6).

**Figure 2:**
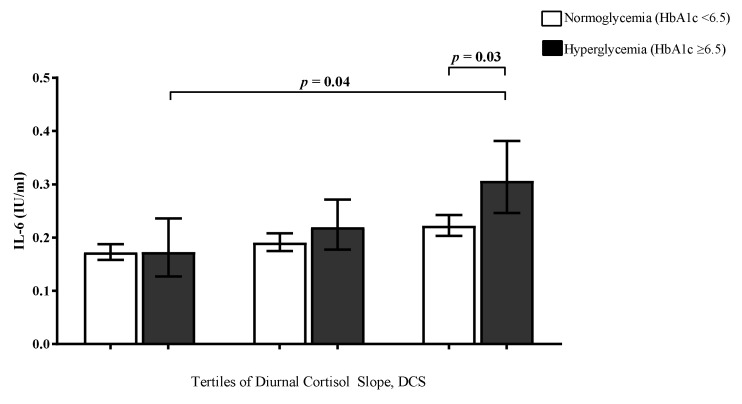
Age and sex-adjusted geometric means of interleukin-6 (IL-6) (pg/mL) levels by diurnal cortisol slope (DCS) tertiles by glycaemia status (glycated haemoglobin, HbA1c cut-off 6.5%) (*n* = 758).

Figure 1 displays the linear association between DCS and IL-6 in the total study population. DCS was negatively associated with IL-6 levels demonstrating that a steeper DCS, a more dynamic DCS, exhibits lower IL-6 levels while a flattened DCS was associated with higher IL-6 concentrations. Next, we analysed the association between DCS tertiles and IL-6 levels stratified by glycaemic status (HbA1c < 6.5% or HbA1c ≥ 6.5%). We found that a flatter DCS was significantly associated with elevated IL-6 levels in the total population (*p* = 0.008) and the effect was strongest in individuals with hyperglycaemia, even after adjusting for age and sex *p* = 0.004). Figure 2 illustrates that IL-6 levels were increased in individuals with hyperglycaemia over the increasing tertiles of the DCS.

The interaction terms of DCS * IL-6 on HbA1c levels were not significant in both crude and fully adjusted models (Model 1: β = 0.03, SE = 0.04, *p* = 0.44; Model 2: β = 0.003, SE = 0.001, *p* = 0.60). The non-significant effect modification by IL-6 levels on the DCS and HbA1c link opens the door to further exploring its potential mediation effect.

### 3.3. Mediation Analysis

**Figure 3:**
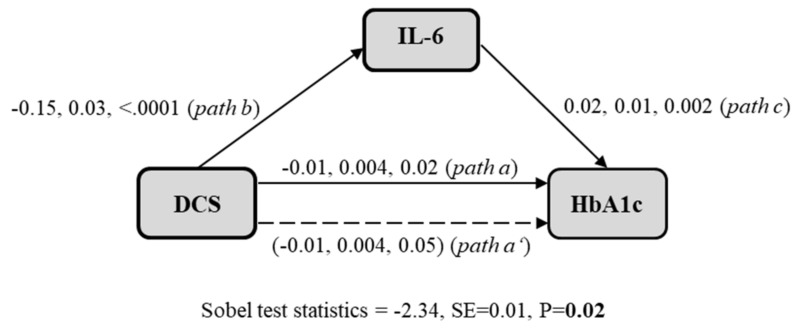
Mediation analysis of chronic inflammation (measured by interleukin-6, IL-6) on the association between diurnal cortisol slope (DCS) and glycaemia (HbA1c). Graphical representation of the mediation analysis: Path a probes the relationship between diurnal cortisol slope (DCS) and glycated haemoglobin (HbA1c) levels. Path b probes the relationship between Interleukin-6 (IL-6) and HbA1c levels, while controlling for cortisol measurements. Path c probes the relationship between DCS and HbA1c. Path c’ probes the relationship between DCS and HbA1c, while controlling for the IL-6 level. The Sobel test was used to test the significance of the mediation effect of IL-6 on the association between DCS and HbA1c levels in age and sex-adjusted regression analyses. Beta estimates with standard deviations and *p*-values are reported for each association examined. The values in parentheses indicate the effect of cortisol on HbA1c when IL-6 is entered into the model.

Within a three-step framework (Figure 3), a multivariate path analytic model (adjusted for age and sex, model 1) investigates an indirect relationship from DCS to glycaemia (HbA1c) via subclinical inflammation (IL-6). The mediating effect was tested by using the Sobel test. First, DCS was associated with HbA1c (in a model not adjusted for IL-6, path a). Second, a flatter DCS was associated with increased IL-6 levels (β = −0.15, SE = 0.03, *p* ≤ 0.0001, path b). Third, we observed a significant association between IL-6 levels and HbA1c, while controlling for DCS measurement (path c). Finally, we observed a weakening of the association between DCS and HbA1c in the presence of the mediating variable (IL-6) (path a’) (Sobel Test Statistic = −2.34, *p* = 0.02). The mediating effect of IL-6 on the association of flatter DCS with HbA1c levels remained even after further adjustment for awakening time, education level, physical activity, alcohol intake, smoking status, and depressive symptoms in Model 2 (*p* from Sobel test = 0.03).

Next, the mediation analysis was then replicated in non-parametric bootstrapping techniques to estimate the indirect effect and proportion mediating effect of IL-6 on the association between DCS and HbA1c. As can be seen in Table 3, the pathway comprising the indirect effect found a significant association between flatter DCS and elevated HbA1c levels (model 1: β = −0.02, 95% CI −0.03–0.01, *p* = 0.01). When further analysed in model 2, the proportion mediated was slightly reduced to 17% (model 2: β = −0.02, 95% CI −0.003–0.001, *p* = 0.01). The mediation analysis revealed that the proportion mediated by IL-6 was 18%, indicating the extent that IL-6 has partly contributed to the indirect relationship between DCS and HbA1c (Table 3). However, further adjusting for BMI reduced the mediating effect of IL-6 on the association between DCS and HBA1c levels to non-significance.

### 3.4. Sensitivity Analyses

In a sensitivity analysis, as opposed to the initial hypothesis, we tested whether the DCS mediates the association between IL-6 and HbA1c. We found no significant mediating effect of DCS on the association between IL-6 and HbA1c (*p* for indirect effect = 0.07, Appendix A.1).

We also repeated the mediation analyses to examine whether IL-6 mediates the association between serum cortisol and HbA1c levels. In the Sobel test, we found that IL-6 mediates the association of serum cortisol with each SD increase in HbA1c level with borderline statistical significance (age- and sex-adjusted *p* value = 0.049) (Appendix A.2). Similar to the DCS finding, further adjusting for BMI reduced the mediating effect of IL-6 on the relationship between cortisol and HbA1c to non-significance (Appendix A.2). The non-parametric bootstrapping mediation analysis revealed that the proportion mediated by IL-6, on the association between serum cortisol and HbA1c levels, did not reach statistical significance (Appendix A.2).

Additional analyses, with adjustments for antidiabetic medications and multimorbiditiy, that includes conditions which are associated with the use of glucocorticoid medications (i.e., chronic obstructive pulmonary disease, rheumatoid arthritis, and gastrointestinal disorders), insulin treatment, and lipid lowering drugs (e.g., Statins), did not alter the observed results (data not shown). Analytical models were also carried out in a dataset without patients undergoing insulin treatment (*n* = 27) or patients with microvascular diabetic complications (e.g., retinopathy, *n* = 4), which might affect inflammation or cortisol secretion. However, the removal of insulin users, or subjects with retinopathy, did not alter the significance of the associations (data not shown).

## 4. Discussion

The present investigation examined the interplay between the diurnal cortisol pattern and subclinical inflammation on HbA1c levels in a sample of 758 community-dwelling older adults. This study reaffirms the association of dysregulated cortisol secretion patterns and hyperglycaemia. We found that a flattened diurnal cortisol slope (DCS)—which was mainly driven by a substantially low morning after awakening cortisol level and a high LNSC level, leading to a blunted cortisol secretion pattern—was significantly associated with increased HbA1c levels. To the best of our knowledge, this study established, for the first time, by employing a path-analysis model, that subclinical inflammation contributed significantly to this association.

### 4.1. Association between Flatter Diurnal Cortisol Slopes and Hyperglycemia

Firstly, the present study provides evidence on a significant association between flattened diurnal cortisol slopes and impaired glucose regulation, confirming previous studies that utilized HbA1c measurement, the KORA-Age study (*n* = 757, mean age = 75 years) [11], the MESA study (*n* = 850, mean age = 70 years) [12], as well as in type 2 diabetes participants [20,21,22]. Controversies still remain of whether the dysregulated cortisol secretion reflects an enhanced or a blunted cortisol reactivity. In sensitivity analyses, we found that a flattened DCS was related to lower morning cortisol levels, higher late-night levels, and a lower overall cortisol output leading to blunted cortisol secretion patterns (see Appendix A.3). Our results confirmed previous population-based studies, which have characterized the flattened diurnal profile as a hypoactive HPA-activation in the general population [23] and among men [22]. However, our data could not show an association between total AUC and HbA1c levels (data not shown). Previous studies demonstrated that cortisol was elevated throughout the day in type 2 diabetes participants of a multi-ethnic population [12] and among women with diabetes in the MESA study [22]. In contrast, men with diabetes in the MESA study displayed a trend of lower total AUC [22]. Similarly, in a clinical study, T2DM patients showed blunted HPA axis reactivity with lower overall AUC than controls, albeit the non-statistical significance [24]. Interestingly, higher total cortisol AUC was associated with lower BMI or waist circumference, especially in those with normal fasting glucose [25], suggesting that a higher total AUC does not necessarily reflect impaired metabolic functioning.

### 4.2. Association of Flatter Diurnal Cortisol Slopes and Subclinical Inflammation

Next, our data show that a flattened DCS is associated with higher IL-6 levels confirming two population-based cohort studies [8,26]. In the MESA study (*n* = 869, mean age = 70 years), a less steep diurnal cortisol decline is associated with higher levels of inflammatory markers (IL-6, IL-10 and CRP) [8]. Similarly, the MIDUS study (*n* = 799, 34–84 years) demonstrated that a flattened diurnal slope is associated with greater concurrent inflammation risk burden at follow-up [26]. In accordance with the role of cortisol as a potent immune suppressant, our findings of a flattened DCS, associated with a blunted cortisol response, generally support the notion that an insufficient GC secretion fails to inhibit the heightened inflammation [7]. Thus, dysregulation of diurnal cortisol, manifested in a hyposecretion pattern, could be related to increased inflammatory activation. However, one should keep in mind, in some studies, a flattened DCS was associated with a greater total cortisol output or hypercortisolism (8,23), while this was not observed in other studies [27,28,29].

### 4.3. Association of Subclinical Inflammation and Hyperglycemia

The present investigation also confirms the well-studied relationship between subclinical inflammation and hyperglycaemia [10]. In our analysis, circulating levels of IL-6 were positively associated with HbA1c levels, which corroborates results from the KORA S4 study (*n* = 850, mean age = 55–74) [30]. This was expected as subclinical inflammation has been shown to precede and be a risk factor of future development of type 2 diabetes [10], and lifestyle modifications and medical treatment attenuating the inflammatory state reduce the risk of future development of type 2 diabetes, suggesting the aetiological role of inflammation in the pathogenesis of type 2 diabetes and associated complications.

### 4.4. Association of Flatter Diurnal Cortisol Slopes and Hyperglycemia Is Partially Mediated by Subclinical Inflammation

As an intermediate result, the present investigation, up to now, has strengthened earlier findings that a flattened diurnal cortisol slope is associated with impaired glucose regulation and also with heightened inflammation. We found evidence that the flattened DCS was associated with a lower overall cortisol output, leading to a blunted cortisol secretion pattern. Guided by a mediation analysis, we were able to show, as a major new finding, that on the pathway between a dysregulated stress-induced cortisol secretion pattern as exposure condition and sustained impaired glucose regulation (evidenced by increased HbA1c levels) as an outcome, and chronic subclinical inflammation (measured as Il-6) substantially mediated this association. We also performed additional analyses to investigate potential reverse causality and found that the data of the present investigation did not support an alternative path with inflammation as exposure and cortisol as mediator.

To the best of our knowledge, no study has assessed the mediating role of subclinical inflammation in the link between a dysregulated cortisol secretion pattern and impaired glucose metabolism so far. Indirect evidence, however, comes from the Whitehall II Study (*n* = 4638; 39–63 years), which assessed the association between psychosocial stress (as a potential cause for the cortisol secretion pattern) and type 2 diabetes and confirmed that this association was weakly mediated by IL-6 [31]. In contrast, a study in the US Hispanic population (*n* = 3923, age = 18–74 years) [32], which measured inflammation by CRP did not evidence a mediation link between chronic psychosocial stress and hyperglycaemia. Interestingly, a study by Lehrke et al. found that cortisol was more superior to IL-6 in predicting insulin resistance after an acute intervention of cardiac surgery in non-diabetic patients [33], suggesting the dominating role of cortisol, compared to inflammatory markers, in stress-dependent insulin resistance.

Our data suggest that impaired diurnal cortisol slopes may be related to the “stress-related circadian dysregulation” (SCiD) initiated and maintained by psychosocial stress [6,34] and pointing to alterations in circadian regulations of the HPA axis, which might result in counter-regulatory responses of stimulated immune cells and consequent downregulation of the expression, function, or both of glucocorticoid receptors, rendering immune cells insensitive to GC, also termed GC resistance [35].

Long-term activation of HPA axis is associated with decreased diurnal variability of cortisol [6]. It has been noted in individuals with “burned-out” diurnal cortisol secretion (including lower awakening cortisol and flatter slope), total cortisol secretion was lower compared to individuals with a more robust high variability diurnal slope, as also seen in individuals with obesity [25,36]. Thus, flattened DCS or low daily cortisol variability may reflect a prior over-stimulated HPA axis, and previously high cortisol levels may have stimulated metabolic disturbances [36]. Because our study was cross-sectional, we cannot confirm a temporal sequence.

It is interesting to note that further adjusting for BMI reduced the effect of IL-6 substantially, which demonstrates that BMI carries the most substantial impact among the covariates under investigation, in diminishing the mediating effect of IL-6 on cortisol measurements and HbA1c levels (*p* indirect effect after BMI adjustment > 0.05, data not shown). Apparently, this could be due to the ‘ceiling effect’ of obesity whereby a cortisol-related association is more strongly observed in lean type 2 diabetes patients than in their obese counterparts.

### 4.5. Strengths and Limitations

The present study collected salivary cortisol measures from a large sample of older community-dwelling of men and women with a high response rate and a very strict quality assessment. The homogeneous and extended dataset allows for a robust adjustment for a set of covariates. This study used HbA1c as an indicator of hyperglycaemia and a proxy for elevated mean glucose levels over the past 2–3 months. HbA1c is also a specific biomarker of severity for impaired glucose metabolism and increasingly accepted as part of type 2 diabetes diagnosis. The use of HbA1c in addition to type 2 diabetes diagnosis enables the identification of undetected type 2 diabetes cases, pre-diabetes state and poorly managed type 2 diabetes. In the present investigation, 82% (*n* = 67) out of 136 individuals who were diagnosed with type 2 diabetes and currently under treatment, had an HbA1c ≥ 6.5%, whereas 18.3% (*n* = 69) were well-controlled with an HbA1c < 6.5%.

We analysed multiple biological system associations in a cross-sectional study design. Although cross-sectional results preclude us from making causal conclusions, the mediation analysis approach has opened the door to analytical tests of the complex interaction of cortisol, subclinical inflammation and hyperglycaemia. Furthermore, a significant mediating effect of cortisol on the association between IL-6 and HbA1c levels was not found in the present study; thus, making potential reverse causality less likely.

The study is limited due to the saliva sample collection on one single day. We observed an individual variability in the time of awakening, but 90.4% of the subjects collected the first sample between 5 and 8 am. We also cannot exclude that the time point of peak cortisol secretion was missed by this procedure. The DCS is restricted to two measurements, and the results are approximations of actual diurnal slope values. However, previous studies have employed similar DCS measures, which were shown to be a robust indicator of diurnal cortisol rhythms [11]. The study cohort is representative for a middle European population of Caucasian ancestry and may not be generalizable to other populations.

## 5. Conclusions

Psychological stress exposure during a lifetime may lead to dysregulated HPA axis reactivity and subsequent impaired glucocorticoid signalling, contributing to the development type 2 diabetes. Here, the present study suggests for a relevant role of IL6 as a mediator between the initial HPA axis dysregulation and glucose metabolism link in old age. Routine assessment of non-invasive salivary cortisol measurements, in addition to inflammatory biomarkers, may allow for a better understanding of the underlying disease conditions and optimisation of the diabetes management beyond targeting glucose control alone. To this end, our findings have addressed the importance of integrating inflammatory state and deregulated HPA axis activation assessments on hyperglycaemia, likely to govern widespread implications for understanding the progression towards type 2 diabetes.

## Figures and Tables

**Table 1:** Characteristics of the KORA Age study population stratified by glycemia status (HbA1c ≥ 6.5% vs. HbA1c < 6.5%) in means (±SD) or N (%) (*n* = 758).

	Hyperglycemia (*n* = 82, 10.8%)	Normoglycemia (*n* = 676, 89.2%)	Overall *	Men *	Women *
**Biomarkers °**					
HbA1c (mmol/mol)	53.2 (±6.0)	37.4 (±3.6)	<0.0001	<0.0001	<0.0001
DCS	1.5 (±0.8)	1.7 (±0.8)	0.04	0.19	0.10
Cortisol_AUC_ (*n* = 734)	23.4 (±14.3)	22.7 (±12.5)	0.78	0.14	0.32
IL-6 (IU/mL)	0.3 (±0.2)	0.2 (±0.3)	0.01	0.27	0.02
Serum cortisol (µg/dL)	9.53 (±3.4)	9.4 (±4.0)	0.78	0.43	0.47
Sociodemographics *					
Mean age (SD)	75.8 (±5.7)	75.0 (±6.3)	0.19	0.85	0.07
Female	33 (40.2)	335 (49.6)	0.11	-	-
Low education	66 (80.5)	482 (71.3)	0.08	0.07	0.16
Living alone	53 (65.4)	440 (65.4)	0.99	0.83	0.53
Cardiometabolic risk factors *					
High alcohol intake	61 (74.4)	584 (90.5)	0.20	0.32	0.12
Current smoker	3 (0.4)	28 (4.1)	0.83	0.52	0.23
Physically inactive	43 (52.4)	282 (41.7)	0.06	0.05	0.50
BMI, kg/m^2^	31.6 (±4.6)	28.2 (±4.3)	<0.0001	<0.0001	<0.0001
Total/HDL cholesterol	4.3 (±1.1)	3.9 (±1.0)	0.005	0.008	0.30
Hypertension	70 (85.4)	496 (65.5)	0.02	0.26	0.03
Multimorbidity	71 (86.6)	401 (59.5)	<0.0001	0.004	0.001
Statin use	34 (41.5)	171 (25.3)	0.002	0.03	0.02
Antidiabetic medications	65 (79.3)	17 (20.7)	<0.0001	<0.0001	<0.0001
Psychological factors *					
Depressed mood	1 (1.3)	11 (1.6)	0.80	0.41	0.63
Anxiety	9 (11.4)	45 (6.7)	0.13	0.67	0.07
Sleep problems	37 (45.1)	328 (48.5)	0.72	0.34	0.50

° Age- and/or sex-adjusted least-squared (LS) means (*p*-values). * *p*-value for differences across DCS tertiles; unadjusted *p*-values, *t*-test for continuous variables and chi-square-test for categorical variables. Abbreviatons: Glycated haemoglobin (HbA1c), Diurnal Cortisol Slope (DCS), Interleukin-6 (IL-6), Body-Mass-Index (BMI), High-density lipoprotein (HDL) cholesterol.

**Table 2:** Characteristics of the KORA-Age study population stratified by diurnal cortisol slope (DCS) tertiles (flatter, medium or steeper) in means (±SD) or N (%) (*n* = 758).

	Flatter (32.9%, *n* = 247)	Medium (34.2%, *n* = 257)	Steeper (32.9%, *n* = 247)	Overall	Men	Women
**Biomarkers °**						
DCS	0.80 (±0.5)	2.36 (±2.1)	2.55 (±0.4)	<0.0001	<0.0001	<0.0001
Cortisol _AUC_ (*n* = 734)	19.3 (±11.9)	21.2 (±9.7)	27.7 (±14.5)	<0.0001	<0.0001	<0.0001
Serum cortisol (µg/dL)	9.6 (±4.1)	9.4 (±4.1)	9.3 (±3.7)	0.49	0.92	0.46
IL-6 (IU/mL)	0.3 (±0.3)	0.2 (±0.2)	0.2 (±0.2)	<0.0001	<0.00001	0.002
HbA1c (mmol/mol)	39.8 (±6.7)	39.2 (±6.4)	38.4 (±5.5)	0.04	0.29	0.15
Sociodemographics *						
Mean age (SD)	76.1 (±6.2)	75.1 (±6.2)	74.0 (±6.2)	0.009	0.56	<0.0001
Female	120 (33.0)	124 (34.1)	120 (33.0)	0.99	-	-
Low education	177 (71.7)	182 (70.8)	183 (74.1)	0.70	0.60	0.90
Living alone	91 (36.8)	89 (35.2)	78 (31.6)	0.45	0.98	0.18
Cardiometabolic risk factors *						
High Alcohol Intake	75 (30.1)	94 (36.7)	70 (28.3)	0.20	0.32	0.12
Current smoker	13 (5.3)	10 (3.9)	8 (3.2)	0.51	0.75	0.60
Physically inactive	120 (49.6)	107 (41.6)	96 (38.9)	0.08	0.81	0.04
BMI, kg/m^2^	28.9 (±4.7)	28.7 (±4.3)	28.1 (±4.1)	0.27	0.98	0.10
Total/HDL cholesterol	4.0 (±1.1)	3.9 (±0.9)	4.0 (±1.0)	0.96	0.52	0.22
Type 2 diabetes	44 (17.8)	46 (17.9)	43 (17.4)	0.93	0.71	0.55
Antidiabetic medication	40 (37.7)	36 (34.0)	30 (28.3)	0.48	0.90	0.43
Statin use	79 (38.5)	70 (34.2)	56 (27.3)	0.09	0.03	0.87
Hypertension	181 (73.3)	195 (75.9)	183 (74.4)	0.80	0.66	0.18
Multimorbidity	170 (69.1)	153 (59.5)	143 (58.1)	0.11	0.02	0.78
Psychological factors *						
Depressed mood	4 (1.7)	5 (2.0)	3 (1.2)	0.80	0.79	0.37
Anxiety	19 (7.8)	19 (7.5)	15 (6.1)	0.73	0.81	0.88
Sleep problems	116 (47.5)	123 (48.8)	124 (50.4)	0.82	0.45	0.87

° Age- and/or sex-adjusted least-squared (LS) means (*p*-values) calculated from GLM models (reference category = steep DCS). * *p*-value for differences across DCS tertiles; unadjusted *p*-values, *t*-test for continuous variables and chi-square-test for categorical variables. Abbreviatons: Glycated haemoglobin (HbA1c), Diurnal Cortisol Slope (DCS), Interleukin-6 (IL-6), High-density lipoprotein (HDL) cholesterol.

**Table 3:** Standardized effects (β estimate, 95% CI) and *p*-values of diurnal cortisol slope (DCS) on HbA1c via IL-6 as a mediator, and the proportion of association between cortisol and HbA1c mediated by IL-6 (*n* = 758).

	Direct Effects (DCS → HbA1c)		Indirect Effects (Mediation Effect) DCS → IL−6 → HbA1c		Total Effects		Proportion Mediated	
	β (95% CI)	*p*	β (95% CI)	*p*	β (95% CI)	*p*	β (95% CI)	*p*
**Model 1**	−0.07 (−0.15; −0.001)	0.05	−0.02 (−0.03–−0.01)	**0.01**	−0.10 (−0.16–0.02)	**0.03**	0.18 (0.04–0.82)	**0.03**
**Model 2**	−0.01 (−0.01–0.001)	0.10	−0.02 (−0.003–0.001)	**0.01**	−0.01 (−0.02–0.001)	**0.04**	0.17 (−0.003–1.04)	**0.04**
**Model 3**	−0.01 (−0.01–0.001)	0.09	0.0002 (−0.001–0.001)	0.71	−0.006 (−0.01–0.001)	0.10	−0.02 (−0.62–0.48)	0.75

Model 1: adjusted for age and sex; Model 2: Model 1 + awakening time, education level, physical activity, alcohol intake, smoking and depressed mood; Model 3: Model 2 + Body-Mass-Index (BMI). Bold values denote statistical significance at the *p* < 0.05 level.

**Figure A1:**
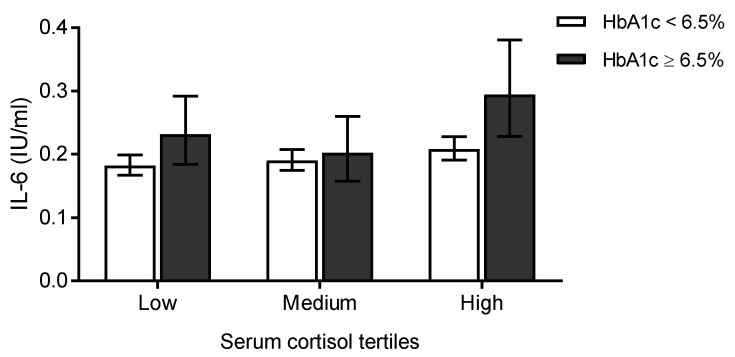
Association of serum cortisol, Interleukin-6 (IL-6) and HbA1c.

**Figure A2:**
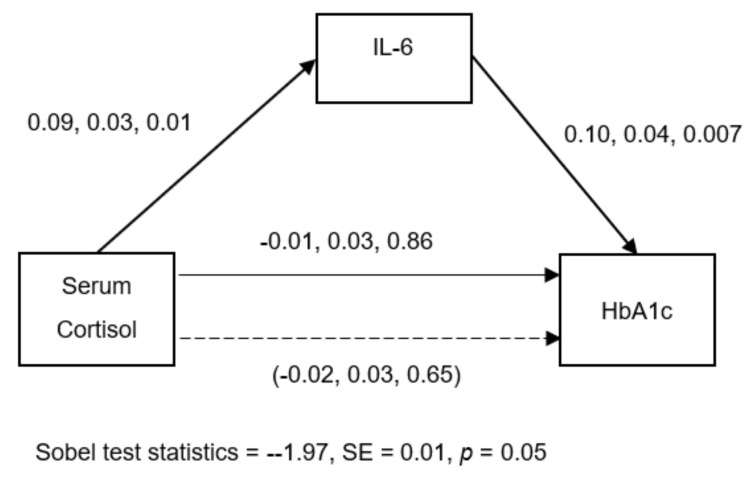
Mediation analysis of chronic inflammation (measured by interleukin-6, IL-6) on the association between serum cortisol levels and glycaemia (HbA1c).

**Figure A3:**
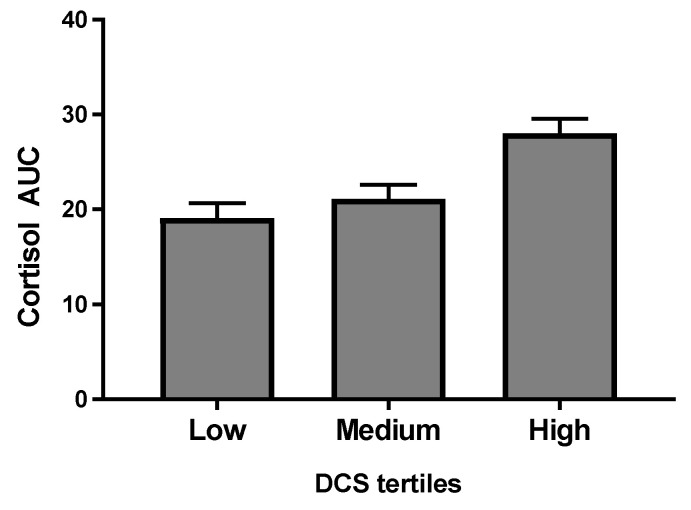
Age and sex-adjusted geometric means of LNSC, morning after awakening (M1) cortisol, and total cortisol output calculated by AUC.

**Figure A4:**
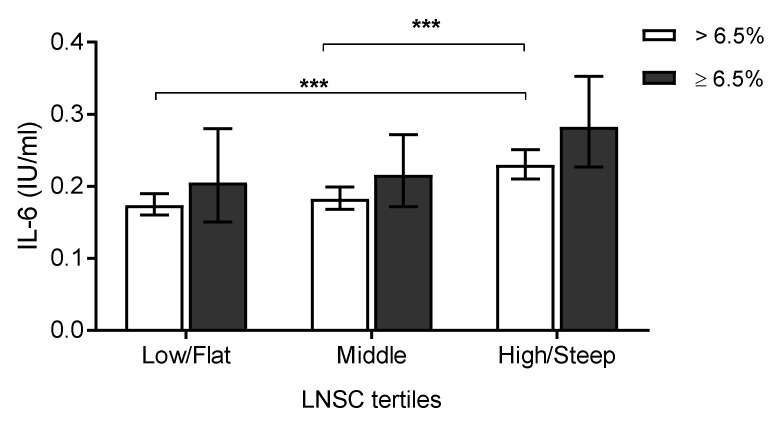
Age- and sex-adjusted LSmeans of Interleukin-6 (IL-6) (pg/mL) concentration by tertiles of LNSC according to glycemia status using the HbA1c cut-off of 6.5% (*n* = 758).

## Data Availability

For approved reasons, access restrictions apply to the data underlying the findings and thus they cannot be made freely available. The data are subject to national data protection laws, and restrictions were imposed by the ethics committee to ensure data privacy of the study participants. However, they can be applied for through an individual project agreement with KORA. Applications for access to the data sets can be found at the following link: https://www.helmholtz-muenchen.de/en/kora/about-kora/kora-today/index.html (access data 21 June 2021).

## Appendix A

Appendix A.1: Standardized effects (β estimate, 95% CI) and *p*-values of IL-6 on HbA1c via diurnal cortisol slope (DCS) as a mediator, and the proportion of association between IL-6 and HbA1c mediated by cortisol (*n* = 758). Appendix A.2: Association of serum cortisol, Interleukin-6 (IL-6) and HbA1c. Appendix A.3: Association of late-night salivary cortisol (LNSC), Interleukin-6 (IL-6) and HbA1c.

### Appendix A.1

**Table A1:** Standardized effects (β estimate, 95% CI) and *p*-values of IL-6 on HbA1c via diurnal cortisol slope (DCS) as a mediator, and the proportion of association between IL-6 and HbA1c mediated by cortisol (*n* = 758).

Direct Effects (IL-6 → HbA1c)		Indirect Effects (Mediation Effect) (IL-6 → DCS → HbA1c)		Total Effects		Proportion Mediated	
β (95% CI)	*p*	β (95% CI)	*p*	β (95% CI)	*p*	β (95% CI)	*p*
−0.001 (−0.01–0.01)	0.79	0.001 (−0.0001–0.001)	0.10	−0.0001 (−0.01–0.01)	0.99	−0.01 (−0.6–3.96)	0.98

Models adjusted for age and sex, awakening time, low education level, physical activity, alcohol intake, smoking and depressed mood.

### Appendix A.2. Association of Serum Cortisol, Interleukin-6 (IL-6) and HbA1c

Appendix A.2 Figure A1 displays age- and sex-adjusted LS means of Interleukin-6 (IL-6) (IU/mL) concentration by tertiles of serum cortisol according to glycemia status using the HbA1c cut-off of 6.5% (*n* = 758). Serum cortisol was not significantly different between glycemic groups. However, individuals in high serum cortisol tertile display elevated levels of IL-6, particularly in hyperglycemic compared to normoglycemic group, albeit non-statistically significant.

Graphical representation of the mediation analysis: Path a probes the relationship between serum cortisol and glycated hemoglobin (HbA1c) levels. Path b probes the relationship between Interleukin-6 (IL-6) and HbA1c levels, while controlling for serum cortisol measurements. Path c probes the relationship between serum cortisol and HbA1c. Path c’ probes the relationship between serum cortisol and HbA1c, while controlling for the IL-6 level. The Sobel test was used to test the significance of the mediation effect of IL-6 on the association between DCS and HbA1c levels in age and sex-adjusted regression analyses. Beta estimates with standard deviations and *p*-values are reported for each association examined. The values in parentheses indicate the effect of serum cortisol on HbA1c when IL-6 is entered into the model.

**Table A2:** Standardized effects of serum cortisol measures on HbA1c via IL-6 as mediator, in age and sex adjusted models (Estimate, 95% CI, *p*-value).

	Direct Effects (Serum Cortisol → HbA1c)		Indirect Effects (Mediation Effect) (Serum Cortisol → IL-6 → HbA1c)		Total Effects		Proportion Mediated	
	β (95% CI)	*p*	β (95% CI)	*p*	β (95% CI)	*p*	β (95% CI)	*p*
Model 1	−0.002 (−0.08–0.06)	0.66	0.001 (0.002–0.02)	0.01	−0.10 (−0.07–0.07)	0.88	−0.10 (−5.10–−5.04)	0.88
Model 2	−0.004 (−0.02–0.01)	0.53	0.002 (−0.0003–0.001)	0.12	−0.002 (−0.02–0.01)	0.72	−0.14 (−4.08–4.04)	0.72
Model 3	0.0001 (−0.002–0.001)	0.93	−0.008 (−0.002–0.01)	0.93	0.0003 (−0.001–0.01)	0.97	−0.0005 (−0.20–1.31)	0.97

Model 1: adjusted for age and sex; Model 2: Model 1+ awakening time, education level, physical activity, alcohol intake, smoking and depressed mood; Model 3: Model 2+ Body-Mass-Index (BMI).

### Appendix A.3. Association of Late-Night Salivary Cortisol (LNSC), Interleukin-6 (IL-6) and HbA1c

Appendix A.3 Figure A3 depicts the age and sex-adjusted geometric means of LNSC, morning after awakening (M1) cortisol, and total cortisol output calculated by AUC. Next, further analyses were only performed in a model that included LNSC as the independent variable due to the significant association between LNSC and HbA1c as reported in our previous publication [11]. LNSC was not significantly different between glycemic groups. However, individuals in high LNSC tertile display elevated levels of IL-6, particularly in hyperglycemic compared to normoglycemic group, albeit non-statistically significant (See Appendix A.3 Figure A4).

Appendix A.3 Figure A4 displays age- and sex-adjusted LSmeans of Interleukin-6 (IL-6) (pg/mL) concentration by tertiles of LNSC according to glycemia status using the HbA1c cut-off of 6.5% (*n* = 758). Table A2 presents the β estimates (95% CI, *p*-value) of LNSC measurements on HbA1c via IL-6 as a mediator, and the proportion of association between cortisol and HbA1c mediated by IL-6 (*n* = 758). Additional analyses revealed that individuals with flatter diurnal cortisol slope (DCS) were more likely to display lower morning after awakening (M1) levels, elevated late-night (LNSC) cortisol levels and lower overall cortisol output (measured by AUC).

**Table A3:** β estimates (95% CI, *p*-value) of late-night salivary cortisol (LNSC) measurement on HbA1c via IL-6 as a mediator, and the proportion of association between cortisol and HbA1c mediated by IL-6 (*n* = 758).

	Direct Effects (LNSC → HbA1c)		Indirect (Mediation Effect (LNSC→IL-6 → HbA1c)		Total Effects		Proportion Mediated	
	β (95% CI)	*p*	β (95% CI)	*p*	β (95% CI)	*p*	β (95% CI)	*p*
Model 1	0.07 (−0.03–0.14)	0.06	0.02 (0.01–0.03)	<0.001	0.08 (0.013–0.15)	0.01	0.20 (0.04–1.06)	0.01
Model 2	0.01 (−0.001–0.02)	0.09	0.02 (0.0001–0.001)	0.04	0.01 (0.004–0.02)	0.04	0.20 (-0.03–1.11)	0.07
Model 3	0.01 (0.001–0.02)	0.03	−0.0003 (−0.002–0.001)	0.67	0.01 (0.001–0.02)	0.04	-0.03 (-0.32–0.20)	0.68

Model 1: adjusted for age and sex; Model 2: Model 1 + awakening time, education level, physical activity, alcohol intake, smoking and depressed mood; Model 3: Model 2 + Body-Mass-Index (BMI).

In multivariable linear regression analyses, there was no significant interaction between LNSC and IL-6 on the association with HbA1c levels (age and sex-adjusted model: β = −0.01, SE = 0.04, *p* = 0.85; fully adjusted model: β = 0.0001, SE = 0.001, *p* = 0.99). As can be seen in Appendix A.3 Table A3, the pathway comprising the indirect effect found a significant association between elevated LNSC and elevated HbA1c levels (model 2: β = 0.02, 95% CI = 0.0001–0.001, *p* = 0.04). However, a significant proportion mediated effect for LNSC was not found (LNSC: β= 0.20, 95% CI= −0.03–1.11, *p* = 0.07). Further adjusting for BMI reduced the strength of mediating effect of IL-6 on the association between DCS or LNSC and HBA1c levels. To test for the potential reverse causality, we further tested whether LNSC mediates the association between IL-6 and HbA1c. There was no signification mediating effect of LNSC on the association between IL-6 and HbA1c (*p* for indirect effect = 0.70).
